# Auriculotherapy associated with Baduanjin Qigong on anxiety and quality of life in university students: a quasi-experimental study

**DOI:** 10.15649/cuidarte.4728

**Published:** 2025-07-17

**Authors:** Quésia Quinto Loreto, Wesley Silva Teixeira, Henrique Andrade Barbosa, Jaqueline D'Paula Ribeiro Vieira Torres, Viviane Carrasco, Hérica Pinheiro Corrêa, Diego Dias de Araújo

**Affiliations:** 1 Universidade Estadual de Montes Claros (UNIMONTES), Montes Claros, Brazil. E-mail: quesialoreto@gmail.com (UNIMONTES) Montes Claros Brazil quesialoreto@gmail.com; 2 Universidade Estadual de Montes Claros (UNIMONTES), Montes Claros, Brazil. E-mail: silva.wesley59@gmail.com (UNIMONTES) Montes Claros Brazil silva.wesley59@gmail.com; 3 Universidade Estadual de Montes Claros (UNIMONTES), Montes Claros, Brazil. E-mail: henriqueabarbosa2007@gmail.com (UNIMONTES) Montes Claros Brazil henriqueabarbosa2007@gmail.com; 4 Universidade Estadual de Montes Claros (UNIMONTES), Montes Claros, Brazil E-mail: jaqueline.vieira@live.com (UNIMONTES) Montes Claros Brazil jaqueline.vieira@live.com; 5 Estadual de Montes Claros (UNIMONTES), Montes Claros, Brazil. E-mail: viviane.carrasco@unimontes.br (UNIMONTES) Montes Claros Brazil viviane.carrasco@unimontes.br; 6 Hospital Cristo Rei, Montalvânia, Brazil. E-mail: hericapc@yahoo.com.br Hospital Cristo Rei Montalvânia Brazil hericapc@yahoo.com.br; 7 Universidade Estadual de Montes Claros (UNIMONTES), Montes Claros, Brazil. E-mail: diego.araujo@unimontes.br (UNIMONTES) Montes Claros Brazil diego.araujo@unimontes.br

**Keywords:** Anxiety, Auriculotherapy, Qigong, Students, Quality of Life, Ansiedad, Auriculoterapia, Qigong, Estudiantes, Calidad de Vida, Ansiedade, Auriculoterapia, Qigong, Estudantes, Qualidade de Vida

## Abstract

**Introduction:**

College students are exposed to internal and external factors that increase the risk of anxiety and can negatively impact their quality of life.

**Objective:**

To evaluate anxiety and quality of life before and after the application of auriculotherapy associated with Baduanjin Qigong and the satisfaction of college students with the treatment.

**Materials and Methods:**

Quasi-experimental study with 44 college students. Four sessions of auriculotherapy and Baduanjin Qigong were carried out, focusing on anxiety and quality of life. The evaluation occurred before and after the treatment using the State-Trait Anxiety Inventory and World Health Organization Quality of Life-Bref instruments. Satisfaction with the intervention was investigated. Descriptive and bivariate analysis was performed.

**Results:**

The sample was composed mainly of: women in the nursing course, with no fixed income and brown skin color. Regarding the final average of the scores evaluated, there was a reduction of 13.77 in anxiety (p<0.001), while in quality of life, there was an increase of 13.88 in the physical domain (p<0.001), 9.10 in the perception of quality of life (p=0.037) and 16.48 in satisfaction with health (p<0.001). Of the participants, 45.50% demonstrated satisfaction with the intervention.

**Discussion:**

The importance of auriculotherapy associated with Baduanjin Qigong as a care to reduce anxiety and prevent changes in quality of life that may affect the well-being of university students is highlighted. Strategies for implementing the intervention are necessary.

**Conclusion:**

The study provides evidence of the positive effect of auriculotherapy associated with Baduanjin Qigong on anxiety and quality of life of university students, impacting health promotion.

## Resumen

**Introducción:**

Los estudiantes universitarios están expuestos a factores internos y externos que aumentan el riesgo de ansiedad y pueden afectar negativamente su calidad de vida.

**Objetivo:**

Evaluar la ansiedad y la calidad de vida antes y después de la aplicación de auriculoterapia asociada a Baduanjin Qigong, así como la satisfacción de los estudiantes universitarios con el tratamiento.

**Materiales y métodos:**

Estudio cuasiexperimental con 44 estudiantes universitarios. Se realizaron cuatro sesiones de auriculoterapia y Baduanjin Qigong, centrándose en la ansiedad y la calidad de vida. La evaluación se realizó antes y después del tratamiento mediante el Inventario de Ansiedad Estado-Rasgo y el instrumento Bref de Calidad de Vida de la Organización Mundial de la Salud. Se investigó la satisfacción con la intervención. Se realizó un análisis descriptivo y bivariado.

**Resultados:**

La muestra estuvo compuesta principalmente por mujeres de enfermería, sin ingresos fijos y de piel morena. Respecto al promedio final de los puntajes evaluados, se observó una reducción de 13.77 en ansiedad (p<0.001), mientras que, en calidad de vida, se observó un aumento de 13.88 en el dominio físico (p<0.001), 9.10 en la percepción de calidad de vida (p=0.037) y 16.48 en satisfacción con la salud (p<0.001). De los participantes, el 45.50% demostró satisfacción con la intervención.

**Discusión:**

Se destaca la importancia de la auriculoterapia asociada a Baduanjin Qigong como cuidado para reducir la ansiedad y prevenir cambios en la calidad de vida que puedan afectar el bienestar de los estudiantes universitarios. Son necesarias estrategias para implementar la intervención.

**Conclusiones:**

El estudio aporta evidencia del efecto positivo de la auriculoterapia asociada a Baduanjin Qigong sobre la ansiedad y calidad de vida de los estudiantes universitarios, impactando en la promoción de la salud.

## Resumo

**Introdução:**

Estudantes universitários estão expostos a fatores internos e externos que aumentam o risco de ansiedade e podem impactar negativamente na qualidade de vida.

**Objetivo:**

Avaliar a ansiedade e a qualidade de vida antes e depois da aplicação de auriculoterapia associada ao Qigong Baduanjin e a satisfação de estudantes universitários com o tratamento.

**Materiais e métodos:**

Estudo quase experimental com 44 estudantes universitários. Realizaram-se quatro sessões de auriculoterapia e Qigong Baduanjin, focadas na ansiedade e qualidade de vida. A avaliação ocorreu antes e depois do tratamento pelos instrumentos Inventário de Ansiedade Traço-Estado e World Health Organization Qualityof Life-Bref. Investigou-se a satisfação com a intervenção. Foi realizada análise descritiva e bivariada.

**Resultados:**

A amostra era composta principalmente por: mulheres do curso de enfermagem, sem renda fixa e cor de pele parda. Quanto a média final dos escores avaliados houve redução de 13,77 na ansiedade (p<0001), já na qualidade de vida, aumento de 13,88 no domínio físico (p<0001), 9,10 na percepção da qualidade de vida (p=0,037) e 16,48 na satisfação com a saúde (p<0001). Dos participantes, 45,50% demonstraram satisfação com a intervenção.

**Discussão:**

Destaca-se a importância da auriculoterapia associada ao Qigong Baduanjin como cuidado para reduzir a ansiedade e prevenir alterações na qualidade de vida que possam afetar o bem-estar de estudantes universitários. Estratégias para implementação da intervenção são necessárias.

**Conclusões:**

O estudo fornece evidências do efeito positivo da auriculoterapia associada ao Qigong Baduanjin sobre a ansiedade e qualidade de vida de estudantes universitários, impactando na promoção da saúde.

## Introduction

College students are exposed to a range of factors that can impact their lives during their undergraduate studies. Entering higher education brings with it new interpersonal relationships, expectations, responsibilities, challenges, distance from family and friends, and an intense study routine. These aspects can lead to a decrease in quality of life, stress, and psychosomatic changes, such as anxiety^1^. 

The nursing diagnosis of anxiety, according to NANDA-International^2^, is an emotional response to a diffuse and non-specific threat, in which the individual anticipates imminent danger. This condition can generate a variety of clinical manifestations, including autonomic responses such as tachycardia and sweating, as well as emotional symptoms such as restlessness, agitation and avoidance. Cognitive changes, such as difficulty concentrating and exaggerated vigilance, are also common, thus requiring a careful approach for effective symptom management^2^-^4^. 

Anxiety tends to emerge as a temporary protective mechanism in stressful situations. However, when it occurs at high intensity and persistently, it can negatively affect quality of life and be detrimental to college students^3^. Quality of life is a comprehensive and dynamic concept that can vary according to different sociocultural, environmental, and psychological factors. This perception is individual and subjective, being influenced by the person's position in their life context^5^. The assessment of quality of life involves the person's perception of their health, physical, psychological, social, and environmental aspects^6^. The domains of quality of life that college students may experience the greatest negative impact on are perception of quality of life, social relationships, environment and physical^4^. 

Given the negative impact of anxiety on quality of life, the importance of implementing actions that can help university students manage their mental and emotional state becomes evident, so that they can conduct academic, social and personal activities in a satisfactory and healthy manner^4^. In several countries, auriculotherapy and Baduanjin Qigong have been gaining prominence as effective complementary practices in the academic environment, being widely used to promote the mental and physical well-being of students^7^, which reinforces their importance in the university context. 

Integrative and Complementary Practices (ICPs) are a set of therapeutic practices, as well as resources, with their own diagnostic systems. They contribute to the balance of body and mind, to a good state of physical and emotional health, by adding natural and holistic practices to the health of the individual, treating them in their entirety^8^. 

Among the PICs, auriculotherapy is a Traditional Chinese Medicine (TCM) technique that has been used with proven efficacy in improving anxiety and quality of life^6^,^9^. This practice uses the stimulation of specific points in the ear, for example, through acupressure, moxa and puncture^4^. Through stimulation of the auricular points, responses occur in the Central Nervous System and harmony of the energy channels, with consequent release of hormones, chemical and endogenous substances that can positively influence physical and emotional problems^9^. 

Qigong, in turn, has been studied and has been shown to be a strong resource for controlling anxiety and, consequently, for improving quality of life^10^. It is also an ancient TCM practice, which consists of a set of harmonious body movements combined with breathing. One of its variants is Baduanjin, an aerobic exercise that has demonstrated benefits in reducing stress, anxiety and depression, increasing self-esteem and social support, in addition to significantly improving physical function^7^,^10^. Unlike exercise or resistance training, which focuses primarily on the body, Baduanjin Qigong aims to concentrate and balance the body, mind and soul and achieve the coordinated development of these elements, which can impact style, quality of life and emotions^7^,^10^. 

In this context, the efficacy and safety of auriculotherapy for anxiety and the effect of Baduanjin Qigong on physical and emotional aspects of university students have already been demonstrated in isolation by previous studies^7^,^11^. However, there is a lack of studies that have evaluated the effect of auriculotherapy associated with Baduanjin Qigong on anxiety and quality of life of university students. Given the above, the objective of this study was to evaluate anxiety and quality of life before and after the application of auriculotherapy associated with Baduanjin Qigong and the satisfaction of university students with the treatment. The hypothesis states that there is a significant difference in the level of anxiety and quality of life between students who receive auriculotherapy associated with Baduanjin Qigong, compared to those who do not. 

## Materials and Methods

**Study design**

Quasi-experimental study, with evaluation of a particular group before and after the intervention. It was conducted at a public university in northern Minas Gerais, Brazil, from November 2023 to May 2024. In this design, the researchers applied the auriculotherapy intervention associated with Baduanjin Qigong and observed its effect on the outcome’s “anxiety” and “quality of life”. Subsequently, the satisfaction of university students with the treatment was evaluated. 

**Population and sample; inclusion and exclusion criteria**

The study population consisted of undergraduate university students regularly enrolled in any of the courses at the educational institution where the research was conducted and who presented complaints of symptoms indicative of anxiety. To compose the sample for comparison of means, an error level of 0.05, a confidence level of 95% and an effect size of 0.5 were considered. The interest, accessibility and availability of students to voluntarily participate in the research were considered. 

The inclusion criteria adopted were students aged 18 or over, duly enrolled in a higher education institution, score ≥41 in the State-Trait Anxiety Inventory (STAI)^12^ and with time availability to answer the study questionnaire, as well as to receive the proposed intervention sessions. The STAI anxiety score cutoff point ≥ 41 was adopted based on previous studies, which indicate that this threshold is effective in identifying moderate levels of anxiety, allowing a more accurate selection of participants with significant symptoms for the intervention. The exclusion criteria for participation in the study were: infection, inflammation, injury or deformity in the ear; use of ear piercing (except normal earrings); use of hearing aids; and use of anxiolytic, antidepressant or other psychotropic medications. Students who missed two consecutive sessions or exceeded an interval greater than ten days between them and who did not complete the data collection questionnaire adequately were discontinued from treatment. 

Among the university students, 134 responded to the questionnaire proposed by the research. Of these, three did not meet the score ≥41 in the STAI and 57 were using anxiolytics, antidepressants or other psychotropic medications. Thus, 74 met the inclusion criteria and were invited to participate in the auriculotherapy sessions associated with Baduanjin Qigong. Although 59 university students agreed to participate in the sessions, 44 completed the study protocol (25.40% loss). 

**Study protocol**

During the recruitment of university students, the study and vacancies for auriculotherapy treatment associated with Baduanjin Qigong were advertised on social media, university notice boards and in classrooms. Participants who met the eligibility criteria and agreed to participate in the study signed the Free and Informed Consent Form (FICF). They then answered the sociodemographic and clinical characterization questionnaire and were assessed for anxiety and quality of life (initial assessment). 

A time was then scheduled for them to attend the university's Center for Biological and Health Sciences (CBHS) to undergo the integrative practices of auriculotherapy and Baduanjin Qigong. At the end of the treatment, they were evaluated again for anxiety and quality of life (final assessment) and for satisfaction with the auriculotherapy intervention associated with Baduanjin Qigong. 

The sociodemographic and clinical characterization instrument, adapted from previous studies^5^,^11^, contained the following variables: sex; age; course; period; chronic diseases; medications in use; salary income; family income and skin color. 

Anxiety was assessed using STA^12^. The instrument contains 20 questions, and the sum of the values obtained in each response can vary from 20 to 80 points, so that: from 20 to 40 points is considered a low level of anxiety; from 41 to 60 points a medium level of anxiety and from 60 to 80 points a high level of anxiety. The higher the score, the greater the severity of anxiety, so that in the context, the score value adopted in the present study was 41 points^12^. 

Quality of life was assessed using the World Health Organization Instrument Quality of Life- Bref (WHOQOL- Bref)^13^, an adapted version containing 26 questions covering the physical, social, psychological and environmental domains, in addition to two additional questions assessing the general perception of quality of life and satisfaction with health^14^. The scores for the different domains were calculated according to the guidelines of the WHOQOL-Bref scoring manual^14^. Due to the absence of specific cutoff points for the WHOQOL- Bref , it is considered that the higher the score, the higher the quality of life. Thus, the results were stratified as follows: very good quality of life (81 to 100 points), good quality of life (61 to 80 points), neither good nor bad quality of life (41 to 60 points), poor quality of life (21 to 40 points), and very poor quality of life (0 to 20 points)^13^,^14^. 

Auriculotherapy was performed with radionic crystals, in four sessions, once a week, with alternating ears at each session, for one month. The acupoints applied with a focus on anxiety control were: Shenmen ( TF 4 ), Kidney (CO 10 ), Visceral Nervous System (AH 6 ), Liver (CO 12 ), Spleen (CO 13 ), Heart (CO 15 ), Adrenal (TG 1 ), Subcortex (AT 4 ) and Anxiety (LO 7 )^4^,^9^,^11^. The location of the points was conducted using a point map from the World Federation of Acupuncture Points. Acupuncture Moxibustion Societies^15^. 

Before starting the application of the radionic crystals, the ear was antisepsised with cotton and 70% ethyl alcohol. Then, they were adhered to the ear with microporous tape, with the participant in a seated position. They were instructed not to remove the crystals until the next session. Manual stimulation of the points was not advised due to the risk of injury to the ear and due to stimulation bias. 

Baduanjin Qigong was performed after the application of auriculotherapy. The exercises were practiced in an environment free of noise and sounds, so as not to distract the participants during the exercise. The Baduanjin Qigong exercise program was formulated by the China Sports Bureau and includes 8 movements, namely: “ holding the sky with the palms up to regulate the triple heater”, “ drawing the bow and shooting the arrow”, “separating heaven and earth”, “looking back to prevent the five injuries and seven weaknesses”, “shaking the head and shaking the buttocks”, “raising and lowering with the hands on the heels to strengthen the kidneys”, “clenching the fists and projecting the gaze firmly to increase the Qi ” and “vibrating the back seven times to eliminate the hundred illnesses”^7^,^10^,^16^. 

The interventions were carried out by undergraduate nursing students with prior training in auriculotherapy and training in the Qigong Baduanjin method, in addition to a minimum of one year's experience in the practices of these therapies. 

Finally, the satisfaction of university students with the auriculotherapy intervention associated with Baduanjin Qigong was assessed using an instrument adapted from a previous study^4^. To this end, participants indicated, on a scale of 1 to 5, their degree of satisfaction with the intervention (extremely dissatisfied; dissatisfied; unsure; satisfied; extremely satisfied), the need for the intervention (totally unnecessary; unnecessary; unsure; necessary; totally necessary) and the perception of general health status after the end of treatment (much better; better; no change; worse ; much worse). 

**Analysis of results and statistics**

The data was tabulated and analyzed in the software Statistical Package for the Social Sciences (SPSS) version 24. Continuous variables were analyzed using standard deviation, median, and minimum and maximum values. Categorical variables were analyzed using absolute and relative frequencies. The Shapiro-Wilk test was used to verify data distribution, which showed a normal distribution of scalar variables. To observe the effect of auriculotherapy intervention associated with Baduanjin Qigong on anxiety and quality of life of university students, the Student's t- test was used, considering a significance level of 5% . The full data collected is available for free access and consultation at Mendeley Data^17^. 

**Ethical aspects**

This study complies with the ethical aspects established by the National Health Council of Brazil, which establishes the guidelines and standards for research involving human beings. It was approved by the Research Ethics Committee of the State University of Montes Claros, under protocol CAAE: 73849723.6.0000.5146 and opinion number 6.320.239. All participants signed the informed consent form. 

## Results

Among the 44 research participants, the average age was 22.91 years (SD = 3.92 years), there was a predominance of females (84.10%), brown skin color (45.50%), single (93.20%), nursing students (72.70 %), with a family income of 2 to 3 minimum wages (65.90%), and depression as the main illness (15.90%) ( Table 1).

The STAI scale had an initial meaning among participants of 55.84, classified as a moderate level of anxiety. After the intervention, the final mean was 42.07, observing a statistically significant decrease in the level of anxiety in university students (P <0.001). A statistically significant improvement was also observed in the physical, perception of quality of life and satisfaction with health domains of the WHOQOL- Bref regarding quality of life after treatment with auriculotherapy associated with Baduanjin Qigong ( Table 2).

**Table 1:** Sociodemographic and clinical characterization of university students (n=44), 2024

Variable	n† (%*)
Gender	
Masculine	7 (15.90)
Feminine	37 (84.10)
Age (M ± SD)	22.91±3.92
Skin color	
Brown	20 (45.50)
White	15 (34.10)
Black	9 (20.50)
Marital status	
Single	41 (93.20)
Married	1 (2.30)
Stable union	2 (4.50)
Course	
Nursing	32 (72.70)
Dentistry	2 (4.50)
Physical education	2 (4.50)
Economic sciences	1 (2.30)
Master's degree	1 (2.30)
Right	1 (2.30)
History	1 (2.30)
Social sciences	2 (4.50)
Civil engineering	2 (4.50)
Period	
7th to 9th	23 (52.27)
1st to 3rd	14 (31.80)
4th to 6th	6 (14.63)
10th to 12th	1 (2.40)
Shift	
Integral	36 (81.80)
Nocturnal	7 (15.90)
Daytime	1 (2.30)
Monthly income	
No fixed income	28 (63.63)
1 minimum wage	14 (31.80)
2 to 3 minimum wages	2 (4.50)
Family income	
2 to 3 minimum wages	29 (65.90)
1 minimum wage	6 (13.63)
4 to 5 minimum wages	6 (13.63)
6 to 7 minimum wages	1 (2.30)
7 to 8 minimum wages	2 (4.50)
Housing	
Republic	35 (79.50)
Alone	6 (13.60)
Spouse	2 (4.50)
Children	1 (2.30)
Chronic disease ‡	
Depression	7 (15.90)
Heart disease	1 (2.30)
Other	2 (4.50)

†n – Absolute frequency; *% – Relative frequency; M = Mean; SD = Standard Deviation; ‡The participant could mark more than one answer option.

**Table 2:** Anxiety and Quality of Life Domains before and after auriculotherapy and Baduanjin Qigong (n=44), 2024

STAI and Domains	Pre- intervention (Initial assessment)			Post intervention (Final assessment)			Value - P
	Mean± SD*	Min ‡	Max §	Mean± SD*	Min ‡	Max §	
STAI	55.84±8.87	33.00	75.00	42.07±9.96	24.00	67.00	**<0.001 **
Physical	52.92 ± 12.40	25.00	89.29	66.80 ± 13.17	32.14	100.00	**<0.001 **
Psychological	50.57 ± 15.05	25.00	91.67	54.74 ± 10.15	37.50	83.33	0.054
Social	60.99 ± 18.40	25.00	100.00	67.05 ± 159.97	33.33	100.00	0.094
Environmental	55.47 ± 13.46	18.75	87.50	57.18 ± 13.56	21.88	87.50	0.474
Perception of quality of life	59.66 ± 20.33	0.00	100.00	68.75 ± 18.77	25.00	100.00	**0.037 **
Health satisfaction	44.32 ± 19.34	25.00	75.00	60.80 ± 21.16	0.00	100.00	**<0.001 **

* SD - Standard deviation; Min ‡ - Minimum value; Max § - Maximum value; II Student's t-test for paired samples

Regarding the intervention satisfaction questionnaire, most students demonstrated satisfaction with the intervention (45.50%), assessed the need for the intervention as necessary (45.50%) and improved their perception of their general health status after completing the intervention (54.50%) (Table 3). 

**Table 3:** Satisfaction with the auriculotherapy intervention associated with Baduanjin Qigong (n=44), 2024

Variables	n† (%*)
Satisfaction with the intervention	
Extremely dissatisfied	2 (4.50)
Dissatisfied	0 (0.00)
I am not sure	6 (13.60)
Satisfied	20 (45.50)
Extremely satisfied	16 (36.40)
Perception of the need for intervention	
Totally unnecessary	0 (0.00)
Unnecessary	0 (0.00)
I am not sure	5 (11.40)
Necessary	20 (45.50)
Totally necessary	19 (43.20)
Perception of general health status after completion of the intervention	
Much worse	0 (0.00)
Worse	0 (0.00)
No change	4 (9, 10)
Better	24 (54.50)
Much better	16 (36.40)

†n – Absolute frequency; *% – Relative frequency.

## Discussion

College students tend to present symptoms of anxiety throughout their course, which can negatively influence their quality of life^1^. Therefore, the implementation of interventions to minimize anxiety and the impact of this condition on academic performance and success, as well as on quality of life, are necessary. 

A reduction of 13.77 (p <0.001) was observed in the final average anxiety score. Specifically regarding auriculotherapy, this is an important intervention with proven efficacy in students with mental health outcomes^4^,^18^. Using the STAI scale, a randomized study18 of auriculotherapy, carried out with university students in Germany, showed a 20% decrease in anxiety levels. Regarding Qigong Baduanjin, a study^7^ indicates promising results as a therapy for health control, regarding emotional problems in university students. Of the 39 participants in the intervention group and 34 in the control group, it was observed that in the Qigong Baduanjin group anxiety was significantly reduced compared with the control group (P <.05 - Control group 0.73±0.38, Baduanjin group 0.58±0.45)^7^. Furthermore, other studies show the efficacy of Baduanjin on anxiety, depression, sleep disorders and quality of life in other populations such as adults, children and pre-competition athletes^19^-^21^. 

After treatment with auriculotherapy associated with Baduanjin Qigong, statistically significant results were observed in domains assessed by the WHOQOL- Bref, with emphasis on the 9.10% increase in the perception of quality of life. There was also an increase of 13.88% in the physical domain related to fatigue, pain and quality of sleep; and in the domain of satisfaction with health, with an increase of 16.48%, which denotes a better ability to adopt positive health habits. Similar results regarding quality of life and use of PICs were found in a previous study with university students^4^. 

Regarding the social domain, there was an increase of 6.06% and in the environmental domain an increase of 1.70%, however these domains were not statistically significant. It is believed that these items comprise domains that cannot be modified from an internal and individual perspective, as they are subject to the collective and society^4^. 

The psychological domain increased by 4.17% and remained borderline (p=0.054) in terms of statistical significance. It is emphasized that psychological stress attributed to anxiety can interfere with the quality of life of university students. 

The auriculotherapy protocol adopted in this study was based on three studies^4^,^9^,^11^ who used the auricular points Shenmen, Visceral Nervous System, Subcortex, Kidney, Adrenal, Heart, Spleen, Liver and Anxiety. These points were also used in studies^18^,^22^,^23^ that specifically evaluated anxiety in other populations and contexts. In a randomized investigation with medical students in the context of evaluating the anatomy discipline, anxiety levels were reduced after the intervention with auriculotherapy (p<0.003), the acupoints used were Lung, Shenmen, Kidney, Subcortex, Adrenal Gland^18^. In a randomized, double-blind, controlled and crossover clinical trial, in patients over 18 years of age, who complained of anxiety symptoms, the recommended points were: Sympathetic, Shenmen, Kidney, Heart, Neurasthenia, Liver and Anxiety^24^. The Spleen point was recommended by another randomized clinical trial in lung cancer patients with high levels of anxiety^25^-^28^. 

Regarding the satisfaction of university students with the treatment used, the majority were satisfied, considering the intervention necessary and that their general health status was better after it. Similarly, a study carried out with university students in which the quality of life and satisfaction with auriculotherapy were evaluated in the context of the pandemic highlighted that, specifically, auriculotherapy had high acceptability and rare adverse effects^4^. 

Systematic review summarized evidence on the use of auriculotherapy in the treatment of anxiety in university students. The studies used the STAI to measure anxiety levels and concluded that auriculotherapy contributed to improving or reducing the outcome of^28^. Another randomized clinical trial^20^ investigated the impact of Baduanjin Qigong on depression and insomnia. Although aimed at a population diagnosed with depression, Baduanjin protocols and practices showed promise for emotional and physical symptoms, such as anxiety and sleep quality, reinforcing the value of Qigong in mental health contexts. These studies that used auriculotherapy or Baduanjin Qigong alone showed positive effects in reducing anxiety and improving quality of life, suggesting that the combination of these practices, as performed in this study, can enhance these benefits, expanding the positive impact on the emotional well-being of university students. 

In this context, the findings of the present study are innovative, given the scarcity of previous investigations that evaluated the effect of auriculotherapy associated with Baduanjin Qigong on anxiety and quality of life of university students. The relevance of this type of study in the population mentioned is also highlighted, since they are exposed to internal and external factors that increase the vulnerability to the development of anxiety and decreased quality of life. 

Despite the positive results, the study's limitations include the sample size and the recruitment of participants by convenience, which may have affected the representativeness of the sample, restricting the participants and, consequently, the generalization of the results to the university population as a whole, since it was carried out in a single geographic and academic setting, not reflecting the diversity of other contexts. Most of the students were enrolled in the undergraduate nursing course, which may have influenced the results and their representativeness of the study. Furthermore, although a validated instrument was used to address anxiety outcome, subjective aspects should be considered and the fact that it is an instrument for tracking the problem. However, despite these limitations, the study has methodological rigor to mitigate biases, and statistically significant results were achieved with this design and sample size. 

The results obtained from this study may contribute to the advancement of knowledge in the health and nursing fields, given the innovative aspect of the research evaluating the effect of auriculotherapy intervention associated with Baduanjin Qigong. It is noteworthy that there is a large literature with strong scientific evidence on auriculotherapy and Baduanjin Qigong separately; however, it is important to recognize that the exact mechanisms of action of these therapies are not yet fully understood and that more research is needed in this area. Auriculotherapy and Baduanjin Qigong are low-cost, simple and fast therapies that allow trained professionals to provide effective treatment, whether individually or collectively. Although they are simple, it is recommended that professionals working in the academic context be trained to use the techniques as instruments capable of helping to reduce anxiety and improve the quality of life of university students. 

## Conclusions

 The present study identified a decrease in anxiety, as indicated by the STAI, and an improvement in the physical domain, perception of quality of life and satisfaction with health in the quality of life, according to the WHOQOL- Bref after the auriculotherapy intervention associated with Baduanjin Qigong. In addition, most participants demonstrated satisfaction with the intervention, considered relevant, with an improvement in their general health status after treatment.

 The results indicate that the auriculotherapy intervention associated with Baduanjin Qigong can impact health promotion, prevention of emotional distress, well-being and quality of life of university students. However, the lack of a control group is considered a limiting factor in the ability to establish causality. Therefore, future investigations with experimental, randomized and randomized designs are recommended, in addition to increasing the sample of participants, including other academic contexts and other complementary practices, aiming to deepen the understanding of the effectiveness of these approaches in promoting mental health and quality of life of university students.

## References

[1] de Cristo  F (2023). Estresse, ansiedade e depressão em calouros de uma faculdade pública no Nordeste, Brasil. Revista Psicologia: Teoria e Prática.

[2] Herdman  TH, Kamitsuru  S, Lopes  CT (2021). Diagnósticos de enfermagem da NANDA-I: definições e classificação - 2021-2023.

[3] Sumiya  A, de Checchi  MHR, Farhat  G, dos Santos  KE, Marcos  VM, Tenani  CF (2022). Auriculoterapia para o controle da ansiedade em estudantes universitários: uma revisão integrativa. Fisioterapia Brasil.

[4] Moura  C de C, Lourenço  BG, Alves  B de O, Assis  BB de, Toledo  LV, Ruela  L de O (2023). Quality of life and satisfaction of students with auriculotherapy in the COVID-19 pandemic: a quasi-experimental study. Rev Bras Enferm.

[5] Fleck  MP, Louzada  S, Xavier  M, Chachamovich  E, Vieira  G, Santos  L (2000). Application of the Portuguese version of the abbreviated instrument of quality life WHOQOL-bref. Revista de Saúde Pública.

[6] Miranda Demenech  L, Gomes Paulitsch  R, Silva da Silva  L, Rodrigues Martins  AC, Neiva-Silva  L, de Carvalho Dumith  S (2023). Determinantes sociais da qualidade de vida entre estudantes de graduação e sua associação com o risco de suicídio. Scientia Medica.

[7] Zhang  Y, Jiang  X (2023). The effect of Baduanjin exercise on the physical and mental health of college students: A randomized controlled trial. Medicine (Baltimore).

[8] Rocha  IR,  Senna  MIB, Oliveira  JS de, Paula  JS de (2023). Práticas Integrativas e Complementares em Saúde: a construção (in)completa da política em um município de grande porte no Brasil. Saúde Debate.

[9] Correa  HP, Moura  CC, Azevedo  C, Bernardes  MFVG, Mata  LRFP da, Chianca  TCM (2020). Effects of auriculotherapy on stress, anxiety and depression in adults and older adults: a systematic review. Rev Esc Enferm USP.

[10] Tedeschi  MRM, Assone  T, Ferreira  M, Souza  KMJ de (2022). Functional fitness and quality of life of elderly Lian Gong, Tai Chi and Qigong practitioners. Acta Paul Enferm.

[11] Chueh  KH,  Chang  CC, Yeh  ML (2018). Effects of auricular acupressure on sleep quality, anxiety, and depressed mood in RN-BSN students with sleep disturbance. J Nurs Res.

[12] Biaggio  AMB, Natalício  L (1979). Manual para o Inventário de Ansiedade Traço-Estado (IDATE).

[13] World Health Organization (December, 1996). WHOQOL-BREF: introduction, administration, scoring and generic version of the assessment: field trial version.

[14] Castro  DFA, Fracolli  LA (2013). Quality of life and health promotion: focusing pregnant women. Mundo Saúde.

[15] World Federation of Acupuncture-Moxibustion Societies (2013). Auricular Acupuncture Point (WFAS STANDARD-002: 2012). WJAM. World J Acupunct Moxibustion.

[16] Malheiros  PC, Vanderlei  AD,  Brum  EHM de (2023). Meditation for stress and anxiety relief in undergraduate students: a randomized clinical trial. Rev Bras Educ Med.

[17] de Araújo  DD,  Loreto  QQ,  Silva  WT, Andrade  HB, Torres  JD'PRV, Carrasco  V, Corrêa  HP (2025). Ansiedad y calidad de vida en estudiantes universitarios: estudio cuasiexperimental. Mendeley Data.

[18] Klausenitz  C, Hacker  H, Hesse  T, Kohlmann  T, Endlich  K, Hahnenkamp  K (2016). Auricular acupuncture for exam anxiety in medical students: a randomized crossover investigation. PLoS One.

[19] Rong  J, Li  J, Jing  F,  Ren  Y, Xiao  Y, Pan  Q (2021). Efficacy of Baduanjin exercise for rehabilitation after COVID-19: A protocol for systematic review and meta-analysis. Medicine (Baltimore).

[20] Fan  J, Qian  F, Wang  Q, Chen  B, Wang  L (2021). Efficacy and safety of Qigong Baduanjin exercise in the treatment of depression with insomnia: a randomized controlled study protocol. Medicine (Baltimore).

[21] Rodrigues  JM, Lopes  L,  Gonçalves  M, Machado  JP (2021). Taijiquan and qigong as a mindfulness cognitive-behavioural based therapy on the treatment of cothymia in school-age children: A preliminary study. Journal of Bodywork and Movement Therapies.

[22] Dellovo  AG, Souza  LMA, de Oliveira  JS, Amorim  KS, Groppo  FC (2019). Effects of auriculotherapy and midazolam for anxiety control in patients submitted to third molar extraction. International Journal of Oral and Maxillofacial Surgery.

[23] Lin  L,  Zhang  Y,  Qian  HY, Xu  JL, Xie  CY, Dong  B (2019). Auricular acupressure for cancer-related fatigue during lung cancer chemotherapy: a randomized trial. BMJ Supportive & Palliative Care.

[24] Guo  L,  Liu  Z, Yuan  W (2022). The effect of Baduanjin on the balancing ability of older adults: A systematic review and meta-analysis. Front Med (Lausanne).

[25] Artioli  DP,  Tavares  AL de F, Bertolini  GRF (2019). Auriculotherapy: neurophysiology, points to choose, indications and results on musculoskeletal pain conditions: a systematic review of reviews. BrJP.

[26] Neves  ML (2020). Acupuntura auricular e neuromodulação.

[27] de Paula Xavier  VA, Berberi  I, Miranda  TL (2022). Auriculoterapia: instrumento alternativo no cuidado da ansiedade. Faculdade Sant'Ana em Revista.

[28] Silva  L dos S,  Souza  CC de, Moura  C de C, Andrade  JV de, Azevedo  C,  Silva  LS da (2021). Auriculoterapia para tratamento da ansiedade em estudantes universitários: revisão sistemática. Revista Eletrônica Acervo Saúde.

